# High-Sensitivity Curvature Fiber Sensor Based on Miniature Two-Path Mach–Zehnder Interferometer

**DOI:** 10.3390/mi15080963

**Published:** 2024-07-28

**Authors:** Yue Wu, Yu Liu, Haoran Zhuang, Juan Cao, Yongjie Yang, Xiaojun Zhu, Dan Sun, Yuechun Shi, Rumao Tao

**Affiliations:** 1 School of Information Science and Technology, Nantong University, Nantong 226019, China; 2230310007@stmail.ntu.edu.cn (Y.W.); 2230320007@stmail.ntu.edu.cn (Y.L.); 2330310026@stmail.ntu.edu.cn (H.Z.); cj@ntu.edu.cn (J.C.); yang.yj@ntu.edu.cn (Y.Y.); 2 School of Microelectronics, Nantong University, Nantong 226019, China; 3 Peking University Yangtze Delta Institute of Optoelectronics, Nantong 226019, China; sundan@ydioe.pku.edu.cn; 4 Yongjiang Laboratory, Ningbo 315202, China; yuechun-shi@ylab.ylab.ac.cn; 5 Laser Fusion Research Center, China Academy of Engineering Physics, Mianyang 621900, China

**Keywords:** curvature sensing, miniature two-path, Mach–Zehnder interferometer

## Abstract

This paper introduces a new high-sensitivity curvature fiber sensor based on a miniature two-path Mach–Zehnder interferometer (MTP-MZI). The sensor is fabricated by coupling and fusing the multimode fiber (MMF) with the single-mode fiber (SMF) using arc fusion technology (AFT), resulting in a centimeter-level two-path MZI structure. The sensor represents an innovative approach to MZI coupling technology, which reduces device size, simplifies manufacturing, and lowers costs. In curvature-sensing experiments, the MTP-MZI sensor achieves a maximum curvature sensitivity of −96.70 dB/m^−1^ in the curvature range of 0.0418 m^−1^ to 0.0888 m^−1^, which is an extremely high sensitivity among intensity-modulated curvature sensors. Additionally, temperature-sensing measurements of the MTP-MZI sensor show a maximum temperature sensitivity of 212 pm/°C in the range of 30 °C to 70 °C, which is low temperature sensitivity and solves the cross-sensitivity problem. Thanks to the miniature two-path structure of the MTP-MZI, it provides a new perspective for developing multidimensional and multiparameter measurement methods in MZI fiber sensors.

## 1. Introduction

Fiber optic sensors possess the characteristics of high sensitivity, anti-electromagnetic interference, and small size, which can be extensively employed in diverse domains [1,2,3]. These features render them particularly appropriate for curvature and temperature sensing. In structural health monitoring, fiber optic curvature sensors can play a role in detecting and gauging the deformation of buildings, bridges, and other infrastructure to guarantee their safety and durability [4,5]. Moreover, in biomedical engineering, fiber-optic curvature sensors are utilized to measure body movements, providing valuable data for advanced prosthetics and the monitoring of physical therapy [6,7].

Additionally, fiber optic sensors are widely used for temperature sensing due to their high accuracy and stability. They are employed in various industrial applications to monitor and control temperatures in harsh environments [8,9]. In research settings, fiber optic temperature sensors provide critical data for experiments requiring precise thermal measurements [10]. The versatility of fiber optic sensors in detecting both curvature and temperature makes them invaluable tools across multiple fields.

Over the years, numerous advancements have been made in the development of fiber-optic Mach–Zehnder interferometers (MZIs) for curvature sensing and temperature sensing. Early studies focused on basic single-mode-multimode-single-mode (SMS) fiber structures, which laid the groundwork for more sophisticated designs. For instance, in 2011, Gong et al. developed an all-fiber curvature sensor based on the SMS fiber structure, leveraging the multimode interference effect to generate several notches in the transmitted spectrum, in which the wavelength shifts and intensity changes of these notches varied differently with applied curvature, achieving maximum sensitivities of −10.38 nm/m^−1^ and −130.37 dB/m^−1^, respectively [11]. In 2013, Zhang et al. presented an ultra-high-sensitivity temperature sensor based on a bending SMS structure fixed in a polymer board frame. By changing the curvature of the SMS fiber through the expansion of the polymer board frame, they achieved a sensitivity of 6.5 nm/°C [12]. In 2018, Yang et al. proposed a high-sensitivity curvature sensor using a single-mode-tapered multimode-single-mode (STMS) fiber structure. This design exploits the elastic-optical effect, where bending induces an asymmetry in the light field distribution within the STMS structure, significantly affecting its transmission characteristics. However, it exhibited low-temperature cross-sensitivity [13]. Despite these advancements, traditional MZIs still face significant challenges. One major issue is the limited capability for multiparameter detection, which is crucial for comprehensive monitoring applications. Additionally, the manufacturing processes for these sensors can be complex and costly, hindering their widespread adoption. Addressing these challenges requires innovative approaches that can simplify the manufacturing process, reduce costs, and enhance the functionality of the sensors.

This paper introduces a novel high-sensitivity curvature fiber sensor based on the MTP-MZI. This innovative approach involves coupling and fusing MMF with SMF using arc fusion technology, resulting in a centimeter-level two-path MZI structure. This new method not only reduces the device size and simplifies the manufacturing process but also lowers the cost significantly. The key innovation in our approach is the lateral coupling of SMF and MMF through arc discharge, achieved by positioning the MMF parallel to the SMF in the fusion splicer. This setup ensures efficient excitation of higher-order modes, which is crucial for high-sensitivity sensing. By creating two welding regions, we establish a miniature two-path MZI at the centimeter level, which enhances the sensor’s performance while maintaining a compact form factor. One of the most significant advantages of our proposed MTP-MZI structure is its ability to mitigate cross-sensitivity issues between curvature and temperature. The independent two-path characteristic of the proposed structure ensures that changes in curvature and temperature affect the sensor in distinct ways, allowing for accurate multiparameter detection. This feature is precious in applications where both curvature and temperature need to be monitored simultaneously.

## 2. Working Principle and Fabrication

**Figure 1:**
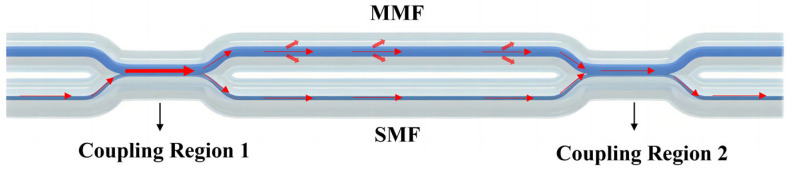
Optical path diagram of the MTP-MZI.

Figure 1 shows the optical path diagram of the MTP-MZI. When light travels from the core of the SMF to coupling region 1, it excites higher-order modes, causing some light to split into the MMF and travel within it, while the rest continues in the SMF. At coupling region 2, the light from the MMF and SMF combines, creating interference, and then continues in the SMF. The distance between the two coupling regions is denoted as L, and it affects the free spectral range (FSR) of the interference spectrum.

The light intensity I of the transmission spectrum in the Mach–Zehnder interferometer (MZI) is given by the following equation:

(1) $$I=I_{0}+I_{m}+2\sqrt{I_{0}I_{m}\;}\cos\Delta \Phi$$

where I_0_ is the fundamental mode intensity, I_m_ is the higher-order mode intensity, ΔΦ is the phase difference between the fundamental and higher-order modes, and ΔΦ can be expressed as follows:

(2) $$\Delta \Phi =\frac{2\pi (n_{\mathrm{eff}}^{01}-n_{\mathrm{eff}}^{\mathrm{mn}})L}{\lambda_{0}}=\frac{2\pi \Delta n_{\mathrm{eff}}^{\mathrm{mn}}L}{\lambda_{0}}$$

where $n_{\mathrm{eff}}^{01}$ and $n_{\mathrm{eff}}^{\mathrm{mn}}$ are the effective refractive indices of the fundamental and higher-order modes, respectively. ${\Delta n}_{\mathrm{eff}}^{\mathrm{mn}}$ is the effective refractive index difference between the fundamental and higher-order modes, L is the interference length, and λ_0_ is the input light wavelength. 

Therefore, when the phase difference satisfies: ΔΦ=(2k+1)π,k=1,2,3…, the transmission intensity reaches a minimum at the following wavelengths:

(3) $$\lambda_{k}^{\mathrm{mn}}=\frac{2\Delta n_{\mathrm{eff}}^{\mathrm{mn}}L}{2k+1}$$

The wavelength interval between two adjacent interference minima in the MZI, known as the FSR, can be expressed as follows:

(4) $$\mathrm{FSR}=|\lambda_{k}^{\mathrm{mn}}-\lambda_{k+1}^{\mathrm{mn}}|=\frac{4\Delta n_{\mathrm{eff}}^{\mathrm{mn}}L}{\left(2k+1)(2k-1\right)}\approx \frac{\lambda_{0}^{2}}{\Delta n_{\mathrm{eff}}^{\mathrm{mn}}L}$$

where λ_0_ is the central wavelength.

In the MTP-MZI, the fringe contrast K of the interference pattern between certain modes can be expressed as follows [14]:

(5) $$K=\frac{2\sqrt{I_{\alpha }I_{\beta }}}{I_{\alpha }+I_{\beta }}$$

where I_α_ and I_β_ indicate the transmission intensities of two interfering beams. The contrast of interference patterns mainly depends on the ratio of intensities between I_α_ and I_β_, as shown in Equation (5). Interference contrast reaches its maximum when the ratio of I_α_ to I_β_ is 1:1. Changes in curvature affect the effective refractive index of both multimode fiber (MMF) and single-mode fiber (SMF) differently, resulting in varying losses for the two modes. Curvature variations also impact the coupling efficiency between the two paths of the MTP-MZI, thereby altering the intensity ratio of I_α_ and I_β_, and subsequently affecting the visibility of interference fringes, including the depth of interference dips. Ultimately, this leads to intensity modulation of the curvature variation in the MTP-MZI.

When the temperature of the sensor changes, its impact on spectral attenuation can be expressed as follows [15]:

(6) $$\Delta \lambda_{T}=\lambda_{i}(\delta +\xi )\Delta T$$

where δ represents the thermal expansion coefficient (TEC), ξ is the thermal optical coefficient (TOC), and ΔT indicates the temperature change. According to Equation (6), it is clear that the temperature sensitivity is determined solely by TEC and TOC, directly affecting wavelength drift. Due to the different operational principles between curvature variation and temperature variation in the MTP-MZI, we employ distinct modulation techniques to measure these parameters. This enhances the sensor’s independence and makes it easier to conduct multiparameter measurements.

**Figure 2:**
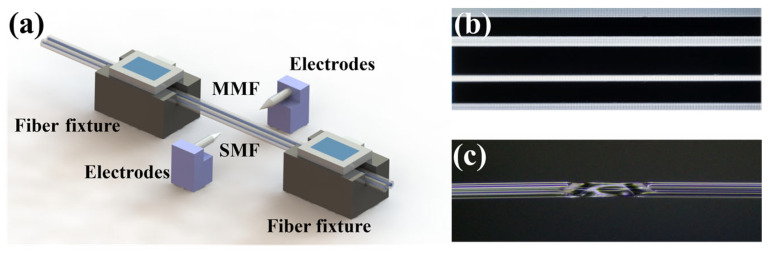
Sensor fabrication diagram. (**a**) Schematic diagram of the preparation process. (**b**) Image displayed by fusion splicer before electrode discharge. (**c**) Detail of the welding region.

The proposed sensor schematic of the fiber fusion splicer is illustrated in Figure 2a. The sensor is primarily made using a commercial fusion splicer. In our experiment, the refractive index (RI) of the core and cladding of the commercial SMF (Corning incorporated, SMF-28e) used were 1.4682 and 1.4629, and the core and cladding diameters were 8.2 μm and 125 μm, respectively. The core and cladding diameters of the commercial MMF (Corning incorporated) were 50 μm and 125 μm, and the corresponding RI are 1.4682 and 1.4629, respectively. Initially, the SMF and MMF have their coatings stripped and are then placed side by side in the fusion splicer. It is important to ensure that they remain in the same plane throughout the process, as shown in Figure 2b. After the fusion splicer automatically stretches the fibers, the discharge mode is set to MM-MM mode with an electrode discharge intensity of 250 a.u. Subsequently, the fusion splicer is switched to manual mode, the discharge area is determined, and a single high-intensity discharge is applied to create a welding region, as shown in Figure 2c. The fibers are then repositioned, placed back in the fusion splicer, and a second high-intensity discharge is performed to create another welding region, successfully fabricating the MTP-MZI.

## 3. Experimental Results and Discussion

**Figure 3:**
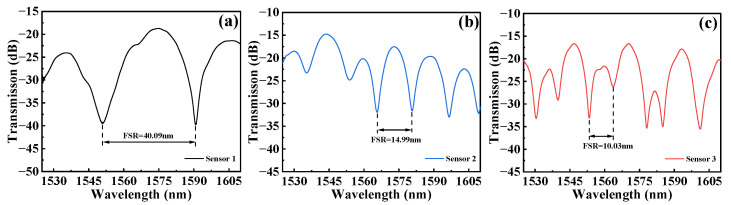
Initial transmission spectra of the three sensors. (**a**) Sensor 1; (**b**) Sensor 2; (**c**) Sensor 3.

In our experimental studies, we fabricated the proposed sensors with three different interference lengths. Sensor 1 has an interference length of 20 mm, Sensor 2 has an interference length of 40 mm, and Sensor 3 has an interference length of 60 mm. Figure 3 shows their respective transmission spectra. From Figure 3, we can see that the number of interference fringes in each sensor’s transmission spectrum changes depending on the interference length, in line with the characteristics of the FSR described in Equation (4). Specifically, FSR decreases as the interference length increases. In our study, for the sensors we tested, FSR changes from 40.09 nm to 10.03 nm as the interference length increases from 20 mm to 60 mm.

**Figure 4:**
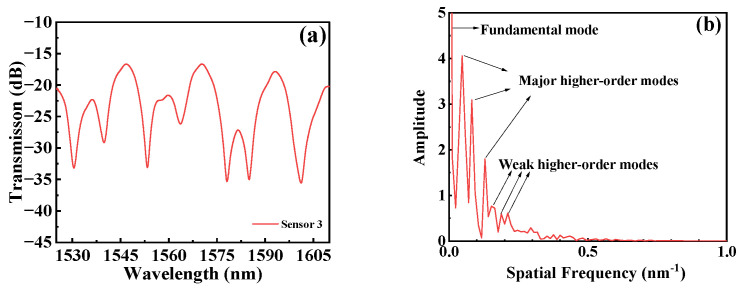
(**a**) Transmission spectrum of Sensor 3; (**b**) spatial frequency spectrum of Sensor 3.

As shown in Figure 4, we performed a fast Fourier transform (FFT) on the transmission spectrum of Sensor 3 to obtain its spatial frequency spectrum. Figure 4b displays three dominant high-order modes and several weak high-order modes that are excited. At the curvature of zero, the intensity of each mode determines the contrast of the interference pattern [16].

**Figure 5:**
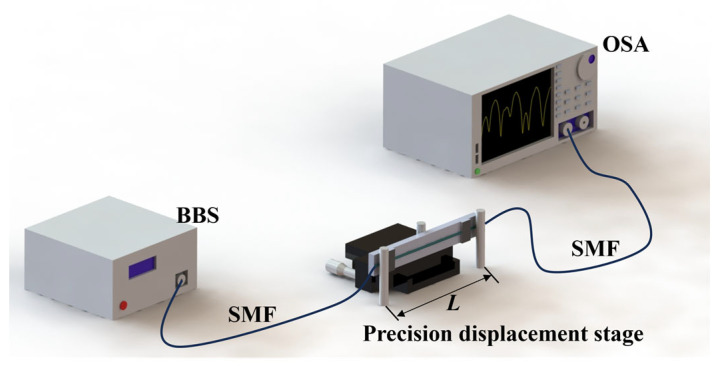
Curvature experimental setup.

We analyzed the curvature sensitivity characteristics of the sensor through experiments, and the experimental setup is shown in Figure 5. The MTP-MZI is mounted on a thin flexible steel ruler and secured to a precise translation stage with a clamp. A micrometer screw slowly controls the stage position by acting exactly on the center of the steel ruler, causing a little deformation in the ruler to ensure accurate control of the MTP-MZI’s curvature. The input port of the sensor is connected to a broadband light source (BBS), and the output port is connected to an optical spectrum analyzer (OSA).

The curvature of the MTP-MZI can be expressed as follows:

(7) $$C=\frac{1}{R}=\sqrt{\frac{2x}{x^{2}+L_{0}^{2}}}$$

where x is the displacement of the precision displacement stage, L_0_ is the distance between fixtures, R is the curvature radius, and C is the curvature.

**Figure 6:**
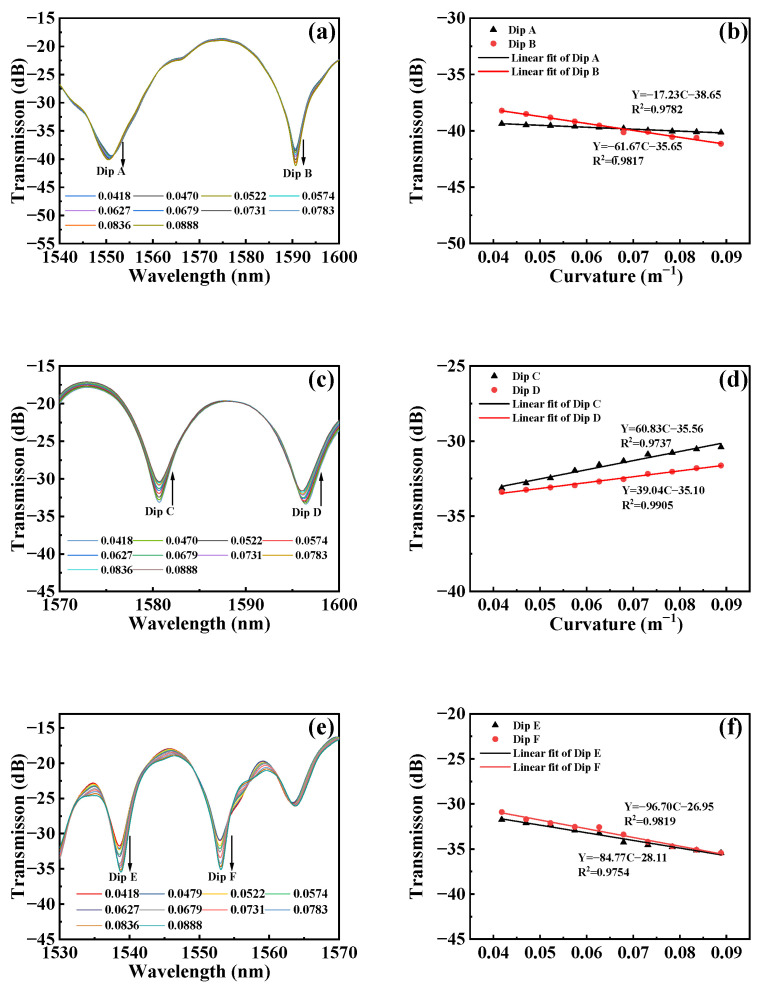
(**a**) Transmission spectra of Sensor 1 at different curvatures. (**b**) Linear fitting of intensity and curvature corresponding to Sensor 1. (**c**) Transmission spectra of Sensor 2 at different curvatures. (**d**) Linear fitting of intensity and curvature corresponding to Sensor 2. (**e**) Transmission spectra of Sensor 3 at different curvatures. (**f**) Linear fitting of intensity and curvature corresponding to Sensor 3.

The response of the three MTP-MZI sensors to changes in curvature is shown in Figure 6. All sensors exhibit intensity modulation when the curvature changes. *In this study, we employed decibels (dB) to assess the curvature measurements of our intensity-modulated fiber MZI sensor due to its good signal strength expression.* For Sensor 1, we studied the dips near 1550 nm and 1590 nm, identified as Dip A and Dip B, respectively. As shown in Figure 6a, within the curvature range of 0.0418 m^−1^ to 0.0888 m^−1^, the intensity of Dip A decreases from −39.38 dB to −40.14 dB, and the intensity of Dip B decreases from −38.22 dB to −41.15 dB. Using linear fitting, the relationship between the dip intensity and curvature for Sensor 1 was determined, as shown in Figure 6b. The curvature sensitivity for Dip A is −17.23 dB/m^−1^ with a correlation coefficient square of 0.9782, while for Dip B, the sensitivity is −67.67 dB/m^−1^ with a correlation coefficient square of 0.9817.

Figure 6c shows the changes in the transmission spectrum of Sensor 2 in the curvature range from 0.0418 m m^−1^ to 0.0888 m^−1^. We analyzed the dips near 1580 nm and 1595 nm, labeled as Dip C and Dip D, respectively. As curvature increases, the intensity of Dip C increases from −33.13 dB to −30.40 dB, and the intensity of Dip D increases from −33.39 dB to −31.624 dB. Figure 6d reflects the linear relationship between the intensity of Dip C and Dip D with curvature. The curvature sensitivity for Dip C reaches 60.83 m^−1^ with a correlation coefficient square of 0.9737, while for Dip D, the sensitivity reaches 39.04 dB/m^−1^ with a correlation coefficient square of 0.9905.

When the curvature is adjusted from 0.0418 m^−1^ to 0.0888 m^−1^, the response of Sensor 3 is shown in Figure 6e. We analyzed the dips near 1538 nm and 1553 nm, labeled as Dip E and Dip F, respectively. As curvature increases, the intensity of Dip E decreases from −31.73 dB to −35.43 dB, and the intensity of Dip F decreases from −30.89 dB to −35.42 dB. Figure 6f shows the linear relationship between the intensity of Dip E and Dip F with curvature. The curvature sensitivity for Dip E reaches −96.70 dB/m^−1^ with a correlation coefficient square of 0.9819, and for Dip F, the sensitivity reaches −84.77 dB/ m^−1^ with a correlation coefficient square of 0.9754.

Figure 6 demonstrates the response characteristics of the three MTP-MZI sensors to curvature changes. By analyzing the changes in light intensity near specific wavelengths in their transmission spectra, we derived the response patterns of each sensor within different curvature ranges. The experimental results show that Sensor 1 exhibits significant intensity modulation near 1550 nm and 1590 nm at Dips A and B, with curvature sensitivities of −17.23 dB/m^−1^ and −67.67 dB/m^−1^, respectively. The transmission spectra of Sensor 2 also show similar intensity modulation at Dips C and D, with curvature sensitivities of 60.83 dB/m^−1^ and 39.04 dB/m^−1^, respectively. Sensor 3 exhibits more pronounced intensity modulation near 1538 nm and 1553 nm at Dips E and F, with curvature sensitivities of −96.70 dB/m^−1^ and −84.77 dB/m^−1^, respectively. These results demonstrate the high sensitivity and stability of the sensors for curvature monitoring, providing reliable technical support for curvature control and monitoring in practical applications.

**Figure 7:**
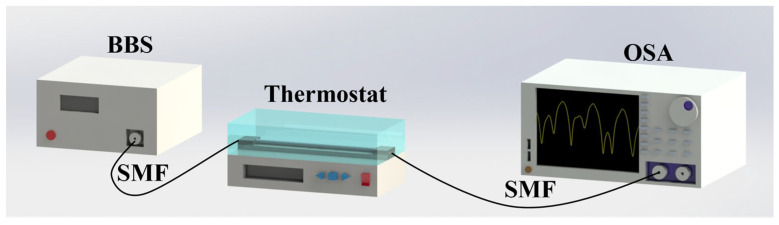
Temperature experimental setup.

To evaluate the performance of the fabricated MTP-MZI sensors under temperature variations, we set up a temperature-sensing experiment, as shown in Figure 7. For this experiment, the MTP-MZI sensor was positioned in a temperature-controlled chamber and connected to the BBS and OSA at each end. The sensor is not subjected to additional stress to ensure the accuracy of the experiment. We then gradually adjusted the temperature of the chamber from 70 °C to 30 °C, collecting data at 5 °C intervals every 10 min during the temperature changes.

**Figure 8:**
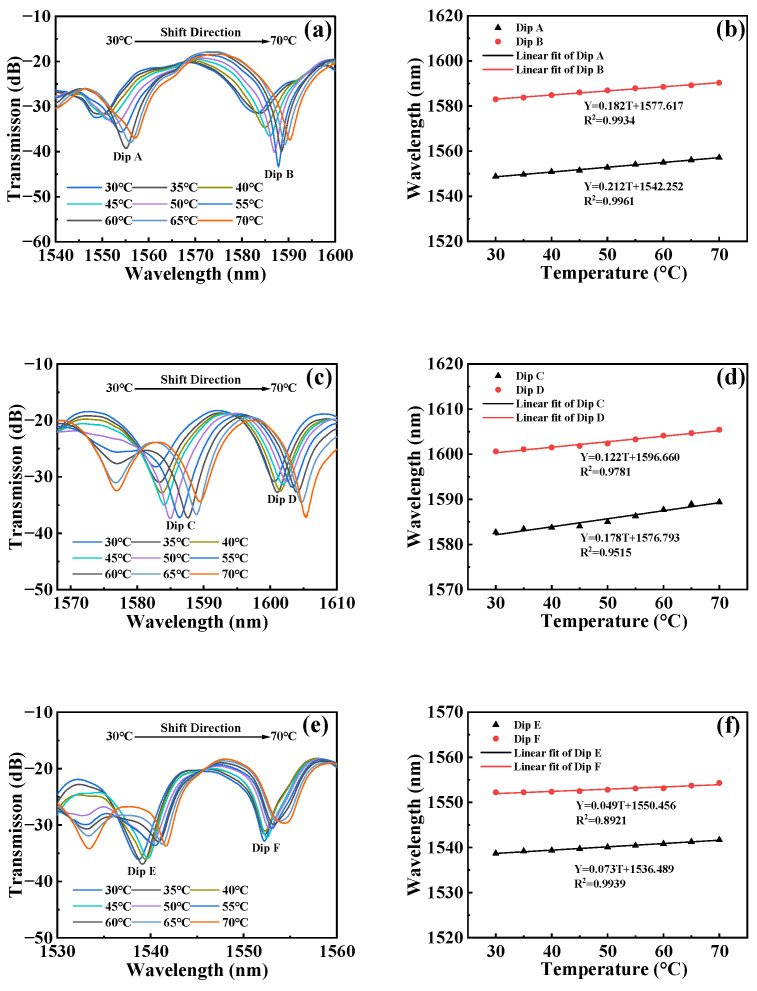
(**a**) Transmission spectra of Sensor 1 at different temperatures. (**b**) Linear fitting of wavelength and temperature corresponding to Sensor 1. (**c**) Transmission spectra of Sensor 2 at different temperatures. (**d**) Linear fitting of wavelength and temperature corresponding to Sensor 2. (**e**) Transmission spectra of Sensor 3 at different temperatures. (**f**) Linear fitting of wavelength and temperature corresponding to Sensor 3.

In Figure 8, we conducted spectral analysis of the sensor at various temperatures using the OSA. After that, we analyzed the temperature response characteristics of the sensor based on this data. In Figure 8a, we observed changes in the transmission spectrum of Sensor 1 with temperature. When the temperature decreased from 70 °C to 30 °C, both Dip A and Dip B exhibited a significant wavelength redshift. Specifically, the wavelength of Dip A shifted from 1557.13 nm to 1548.77 nm, and the wavelength of Dip B shifted from 1590.28 nm to 1582.97 nm. To further explore the relationship between the dip wavelength and temperature for Sensor 1, we used linear fitting. The analysis results, depicted in Figure 8b, revealed that the temperature sensitivity for Dip A is 182 pm/°C with a correlation coefficient square of 0.9934, and for Dip B, the sensitivity is 212 pm/°C with a correlation coefficient square of 0.9961.

The changes in temperature cause the dips for Sensor 2 to shift in wavelength, as illustrated in Figure 8c. When the temperature changes uniformly from 70 °C to 30 °C, the wavelength of Dip C shifts from 1589.37 nm to 1582.72 nm, and the wavelength of Dip D shifts from 1605.41 nm to 1600.62 nm. Upon linear fitting, the temperature sensitivity for Dip C is determined to be 122 pm/°C with a correlation coefficient square of 0.9781, and for Dip D, the sensitivity is 178 pm/°C with a correlation coefficient square of 0.9515, as shown in Figure 8d.

When the temperature changes, the redshift of the dips for Sensor 3 is shown in Figure 8e. The wavelength of Dip E shifts from 1541.69 nm to 1538.69 nm, and the wavelength of Dip F shifts from 1554.30 nm to 1552.23 nm. Figure 8f presents the linear fitting analysis results, indicating that the temperature sensitivity for Dip E is 49 pm/°C with a correlation coefficient square of 0.8921, and for Dip F, the sensitivity is 73 pm/°C with a correlation coefficient square of 0.9939.

Through the temperature-sensing experiments, we obtained the spectral characteristic data of the sensors at different temperatures and performed an in-depth analysis of their temperature response performance. We found that all sensors exhibited wavelength redshift as the temperature gradually decreased. Specifically, for Sensor 1, Dip A and Dip B shifted by 7.7 nm and 7.52 nm, respectively, when the temperature decreased from 70 °C to 30 °C, with temperature sensitivities of 182 pm/°C and 212 pm/°C and high correlation coefficient squares of 0.9934 and 0.9961, respectively. Sensor 2’s Dip C and Dip D and Sensor 3’s Dip E and Dip F also showed similar trends. The results indicate that the proposed MTP-MZI sensors have relatively low temperature sensitivity and good stability.

To further highlight the excellent sensitivity characteristics of the MTP-MZI sensors, we compared them with previously reported fiber sensors, as detailed in Table 1. It is evident from Table 1 that MTP-MZI sensors perform exceptionally well in terms of curvature sensitivity with intensity modulation. Compared to micro- or nanofibers, MTP-MZI sensors are easier to integrate, have simpler fabrication processes, and offer unique advantages in sensing dimensions and scalability.

## 4. Conclusions

In summary, we proposed and demonstrated a fiber optic sensor based on the MTP-MZI. The sensor is fabricated by a simple arc discharge fusion of MMF and SMF. Due to the small size (500 μm) of the welding region prepared by arc discharge, a centimeter-level two-path MZI can be achieved. In terms of curvature detection, the sensor demonstrated a peak curvature sensitivity of −96.70 dB/m^−1^ over an interference length of 60 mm (corresponding to a curvature range of 0.0418 m^−1^ to 0.0888 m^−1^). Meanwhile, the sensor also demonstrated low temperature sensitivity. When the temperature varied from 30 °C to 70 °C, the highest temperature sensitivity was 212 pm/°C. Since temperature sensing shows wavelength shift characteristics and curvature sensing exhibits intensity modulation characteristics, we can address the issue of cross-sensitivity between curvature and temperature by monitoring these two independent parameters. This MTP-MZI sensor provides a new approach for multiparameter measurement and multidimensional detection, with its compact and robust structure offering broad application prospects in structural health monitoring and industrial manufacturing.

## Figures and Tables

**Table 1:** Comparison with previously reported fiber optic sensors.

Parameter	Structure	Range	Sensitivity	Reference
Curvature	SMF-TSMF-SMF	2.706–4.364 m^−1^	51.013 dB/m^−1^	[17]
Curvature	SMF-FMF-SMF	3.96–4.20 m^−1^	−46.13 dB/m^−1^	[18]
Curvature	SMF-SPF-SMF	−0.48–0.48 m^−1^	15.21 dB/m^−1^	[19]
Curvature	The Proposed sensor	0.0418–0.0888 m^−1^	−96.70 dB/m^−1^	**This work**
Temperature	SMF-PCF-SMF	−70–70 °C	1.00 nm/°C	[20]
Temperature	SMF-LTHF-SMF	15–40 °C	1.6 nm/°C	[21]
Temperature	SMF-GRIN-SMF	25–85 °C	0.24 nm/°C	[22]
Temperature	The Proposed sensor	30–70 °C	212 pm/℃	**This work**

## Data Availability

The data presented in this study are available from the corresponding author upon reasonable request.
